# Polygenic Risk Score Prediction for Endometriosis

**DOI:** 10.3389/frph.2021.793226

**Published:** 2021-12-17

**Authors:** Kirstine Kloeve-Mogensen, Palle Duun Rohde, Simone Twisttmann, Marianne Nygaard, Kristina Magaard Koldby, Rudi Steffensen, Christian Møller Dahl, Dorte Rytter, Michael Toft Overgaard, Axel Forman, Lene Christiansen, Mette Nyegaard

**Affiliations:** ^1^Department of Chemistry and Bioscience, Aalborg University, Aalborg, Denmark; ^2^Department of Clinical Immunology, Aalborg University Hospital, Aalborg, Denmark; ^3^Department of Health Science and Technology, Aalborg University, Aalborg, Denmark; ^4^The Danish Twin Registry, Department of Public Health, University of Southern Denmark, Odense, Denmark; ^5^Department of Business and Economics, University of Southern Denmark, Odense, Denmark; ^6^Research Unit for Epidemiology, Department of Public Health, Aarhus University, Aarhus, Denmark; ^7^Department of Gynecology and Obstetrics, Aarhus University Hospital, Skejby, Denmark; ^8^Section of Forensic Genetics, Department of Forensic Medicine, Faculty of Health and Medical Sciences, University of Copenhagen, Copenhagen, Denmark; ^9^Department of Biomedicine, Aarhus University, Aarhus, Denmark

**Keywords:** endometriosis, genetic association, ICD-10, polygenic risk score, subtypes

## Abstract

Endometriosis is a major health care challenge because many young women with endometriosis go undetected for an extended period, which may lead to pain sensitization. Clinical tools to better identify candidates for laparoscopy-guided diagnosis are urgently needed. Since endometriosis has a strong genetic component, there is a growing interest in using genetics as part of the clinical risk assessment. The aim of this work was to investigate the discriminative ability of a polygenic risk score (PRS) for endometriosis using three different cohorts: surgically confirmed cases from the Western Danish endometriosis referral Center (249 cases, 348 controls), cases identified from the Danish Twin Registry (DTR) based on ICD-10 codes from the National Patient Registry (140 cases, 316 controls), and replication analysis in the UK Biobank (2,967 cases, 256,222 controls). Patients with adenomyosis from the DTR (25 cases) and from the UK Biobank (1,883 cases) were included for comparison. The PRS was derived from 14 genetic variants identified in a published genome-wide association study with more than 17,000 cases. The PRS was associated with endometriosis in surgically confirmed cases [odds ratio (OR) = 1.59, *p* = 2.57× 10^−7^] and in cases from the DTR biobank (OR = 1.50, *p* = 0.0001). Combining the two Danish cohorts, each standard deviation increase in PRS was associated with endometriosis (OR = 1.57, *p* = 2.5× 10^−11^), as well as the major subtypes of endometriosis; ovarian (OR = 1.72, *p* = 6.7× 10^−5^), infiltrating (OR = 1.66, *p* = 2.7× 10^−9^), and peritoneal (OR = 1.51, *p* = 2.6 × 10^−3^). These findings were replicated in the UK Biobank with a much larger sample size (OR = 1.28, *p* < 2.2× 10^−16^). The PRS was not associated with adenomyosis, suggesting that adenomyosis is not driven by the same genetic risk variants as endometriosis. Our results suggest that a PRS captures an increased risk of all types of endometriosis rather than an increased risk for endometriosis in specific locations. Although the discriminative accuracy is not yet sufficient as a stand-alone clinical utility, our data demonstrate that genetics risk variants in form of a simple PRS may add significant new discriminatory value. We suggest that an endometriosis PRS in combination with classical clinical risk factors and symptoms could be an important step in developing an urgently needed endometriosis risk stratification tool.

## Introduction

Endometriosis affects 10% of fertile women and represents a major health care challenge (1). Early detection would be of value to avoid sensitization and development of chronic pelvic pain (2, 3). Since laparoscopy is needed for diagnosis, and symptoms overlap with common problems like primary dysmenorrhea (1), a high number of young girls with endometriosis today go undetected. Thus, there is a great need to develop clinical decision tools that will help the medical doctors to better identify cases where laparoscopy is warranted and/or medical treatment can be offered. Despite promising results with different types of circulating molecules (1, 4, 5), biomarkers have so far been of limited value because of modest sample sizes and lack of replication (6).

In recent years, there has been an increasing interest in, including genomic information, risk models. This development is driven by the increasing number of genetic risk variants for common complex diseases being identified through large-scale genome-wide association studies (GWAS) (7, 8), including also studies of endometriosis; a large study investigating association with common variants (9), a smaller study with rare coding variants (10), and a recent study in the Biobank Japan Project (11). Since individual risk loci identified in a GWAS usually only exercise small effect sizes (12–14), aggregating the effects of multiple genetic risk variants into a single score is an area of great interest. One of the most widely used scores is calculated as the sum of risk alleles weighted by single nucleotide polymorphism (SNP) effect sizes derived from GWAS, i.e., a polygenic risk score (PRS) (15, 16). Its correlation with genetic liability has led to widespread use of PRSs in biomedical research (17–19).

The predictive power of a PRS is most optimally studied in clinically well-defined cases, but to increase sample size, studies are also ongoing in large biobanks linked to health registries using phenotypes from for example the International Classification of Diseases (ICD) (20). Some of these efforts include, among others, BioBank Japan (21), FinnGen (22), and the UK Biobank project (23). Although ICD codes are convenient to use, the codes themselves can be suboptimal in defining diseases (24). In Denmark, the Danish National Patient Register (DNPR), containing ICD codes, is one of the world's oldest nationwide hospital registries and is used extensively for research (25), but no systematic studies validating endometriosis diagnoses in this register has been carried out.

The aim of this study was to evaluate the performance of a simple 14-SNP PRS to predict endometriosis in three different settings, namely surgically confirmed endometriosis cases (clinical cohort), cases with endometriosis defined from the Danish Twin Registry biobank (DTR cohort), and replication in the UK Biobank (UKB). The 14 SNPs represent top hits from the largest GWAS metaanalysis of endometriosis published to date (9). The discriminative ability was tested for endometriosis, endometriosis subtypes, and adenomyosis for comparison. Adenomyosis was included because it shares features with endometriosis but is more and more considered its own disease entity (26). The goal was to uncover if genetics could be part of a risk predictor in the future.

## Materials and Methods

### Clinical Cohort

Patients with surgically confirmed endometriosis (*n* = 249) were enrolled from Department of Gynecology and Obstetrics, Aarhus University Hospital, Denmark. All cases were confirmed by laparoscopy, histological examination, and had undergone surgery for endometriosis. The ICD-10 codes for all surgically confirmed cases were extracted from medical records. All had ASRM stages II–IV. The control group consisted of Danish blood donors from the Aalborg University Hospital blood bank, Aalborg, Denmark, and were age-matched women without a diagnose of N80 (*n* = 348). Control samples were anonymized prior to genotyping. The study was approved by The Central Denmark Region Committees on Health Research Ethics (ID 25.736).

### Danish Twin Registry (DTR) Biobank Cohort

Participants from the Danish Twin Registry were drawn from the Danish Twin Registry Infrastructure Study carried out from 2008 to 2011 (27). Unrelated women with an endometriosis diagnosis (N80.1–N80.9) in the Danish National Patient Registry (DNPR) (28) were selected as cases (*n* = 140). Four of the women had a twin sister with endometriosis; these sisters were not included. In addition, unrelated women with a diagnosis of adenomyosis (N80.0) were included for comparison (*n* = 25). Age-matched unrelated women without an N80 diagnose were selected as controls (*n* = 316). Individuals from the Danish Twin Registry were followed in DNPR until March 2014. The study was approved by the Regional Scientific Ethical Committees for Southern Denmark (project ID S-20170120) and registered at Research & Innovation Organization at University of Southern Denmark (registration number 10.584), who approves all scientific projects for University of Southern Denmark according to the Data Protection Regulation.

### UK Biobank

For replication analysis, the 14 SNPs (see section “Assay Design”) were identified from imputed data (best guess genotypes) in the UK Biobank (23). Among unrelated British Caucasian female individuals, cases with endometriosis (N80.1–N80.9) (*n* = 2,967), adenomyosis (N80.0, no N80.1–N80.9) (*n* = 1,883) and controls (no N80 diagnosis, and no self-reported endometriosis) (*n* = 256,222) were identified. ICD-10 codes were identified from main and secondary in-hospital ICD-10 records (data field 41270), while self-reported endometriosis was identified from data field 20002.

### Endometriosis Subtypes From ICD-10 Diagnoses

Four major subgroups of endometriosis were constructed from the ICD-10 N80 diagnoses (Table 1). Cases with multiple different N80 sub-diagnoses were assigned to the most severe category with severity ranked from severe to mild in the following order: N80.5 (endometriosis of intestine), N80.4 (endometriosis of rectovaginal septum and vagina), N80.1 (endometriosis of ovary), N80.3 (endometriosis of pelvic peritoneum), N80.2 (endometriosis of fallopian tube), N80.9 (unspecified), N80.8 (endometriosis of thorax), N80.6 (endometriosis of cutaneous scar). Subjects with adenomyosis only (N80.0) were treated as a separate and distinct category.

**Table 1:** Distribution of endometriosis subtypes and adenomyosis subjects within the three cohorts.

**Disease subtypes**	**Cohorts**		
	**Clinical**	**DTR**	**UK Biobank**
Endometriosis (N80.1-N80.9)	249 (42%)	140 (29%)	2,967 (1.1%)
- infiltrating (N80.4, N80.5)	205 (34%)	5 (1%)	105 (0.04%)
- ovarian (N80.1)	21 (4%)	54 (11%)	1,158 (0.4%)
- peritoneal (N80.2, N80.3)	16 (3%)	44 (9%)	736 (0.3%)
- other (N80.6, N80.8, N80.9)	7 (1%)	37 (8%)	968 (0.4%)
Adenomyosis (N80.0)	0 (0%)	25 (5%)	1,883 (0.7%)

*Percentages in parentheses indicate the case prevalence within each cohort*.

### Assay Design

The 14 genome-wide significant lead SNPs from an endometriosis GWAS comprising 17,045 endometriosis cases and 191,596 controls (9) were initially included for genotyping. One lead SNPs (rs760794) failed assay design, and we instead included rs77294520 which was region-wide associated after conditioning on the index SNP in the *GREB1* locus (Supplementary Table 1). The 14-SNP multiplex genotyping assay was designed as a SNP type assay using the online Fluidigm D3 Assay tool (Supplementary Table 2).

### Genotyping

Genomic DNA was extracted from whole blood using a manual (29) or a semiautomatic (Autopure, Qiagen, Hilden, Germany) salting out method. Genotyping was performed on a Fluidigm Biomark system (Fluidigm Corporation) with the standard SNP genotyping analysis user guide (PN 68000098 Q1), and the genotypes were called using the Fluidigm SNP Genotyping Analysis software, with rigorous manual inspection of all clusters. For quality control, 112 DNA samples were genotyped in duplicate in different runs yielding full (100%) consistency in genotype calls across all duplicates. For each genotyping, chip allele frequencies were compared to the CEU population to exclude allele swop. No significant deviations from Hardy–Weinberg equilibrium were found for any of the SNPs (*p*-value threshold 0.05). Missing genotypes (46 out of in total 15,092 genotypes, i.e., <0.5%) were imputed to the average of the clinical or the biobank cohort sample sets.

### Polygenic Risk Score and Predictive Performance

The 14 SNPs were tested individually for association with endometriosis (N80.1–80.9) using logistic regression, with no covariates and including age-matched women as controls. A PRS for endometriosis was calculated as the weighted sum of risk alleles; $PRS=\;\sum_{i=1}^{14}{\text{X}}_{i}{\hat{b}}_{i}$, where **X**_*i*_ is the *i*-th column of the genotype matrix containing allelic counts of the risk allele (0, 1, or 2) and ${\hat{b}}_{i}$ is the estimated marker effect for the *i*-th variant; here the log odds ratio (OR), which were obtained from (9). The PRS was standardized within each of the three cohorts. For the combined Danish analysis (clinical and DTR biobank cohorts), the PRS was standardized to mean zero and variance 1 in the combined cohort.

Mean PRS between groups were compared using Student's *t*-test, and the discriminative ability of the PRS was determined using area under the receiver operating curve (AUC) and with Nagelkerke's variances explained ($R_{Nag}^{2}$) using the R package qgg (30).

The change in genetic risk with increasing PRS was investigated by binning individuals into 10 groupings according to the decile of the PRS, and the disease prevalence within each bin was determined. For each bin, we furthermore calculated OR using logistic regression with disease status as response variable and decile as predictor with the fifth decile as reference point. The strength of association for the PRS with endometriosis and endometriosis subtypes was estimated by calculating OR per standard deviation increase in standardized PRS.

## Results

### Single Variant Association

An initial single variant association analysis in the two Danish cohorts, the clinical cohort with surgically confirmed endometriosis cases and the DTR biobank cohort with endometriosis cases identified from ICD-10 codes, revealed an overall directional consistency in the effect sizes compared with the discovery GWAS (Supplementary Table 1), indicating that most of the established risk variants also are risk variants in the Danish population.

### PRS Association With Endometriosis

**Figure 1:**
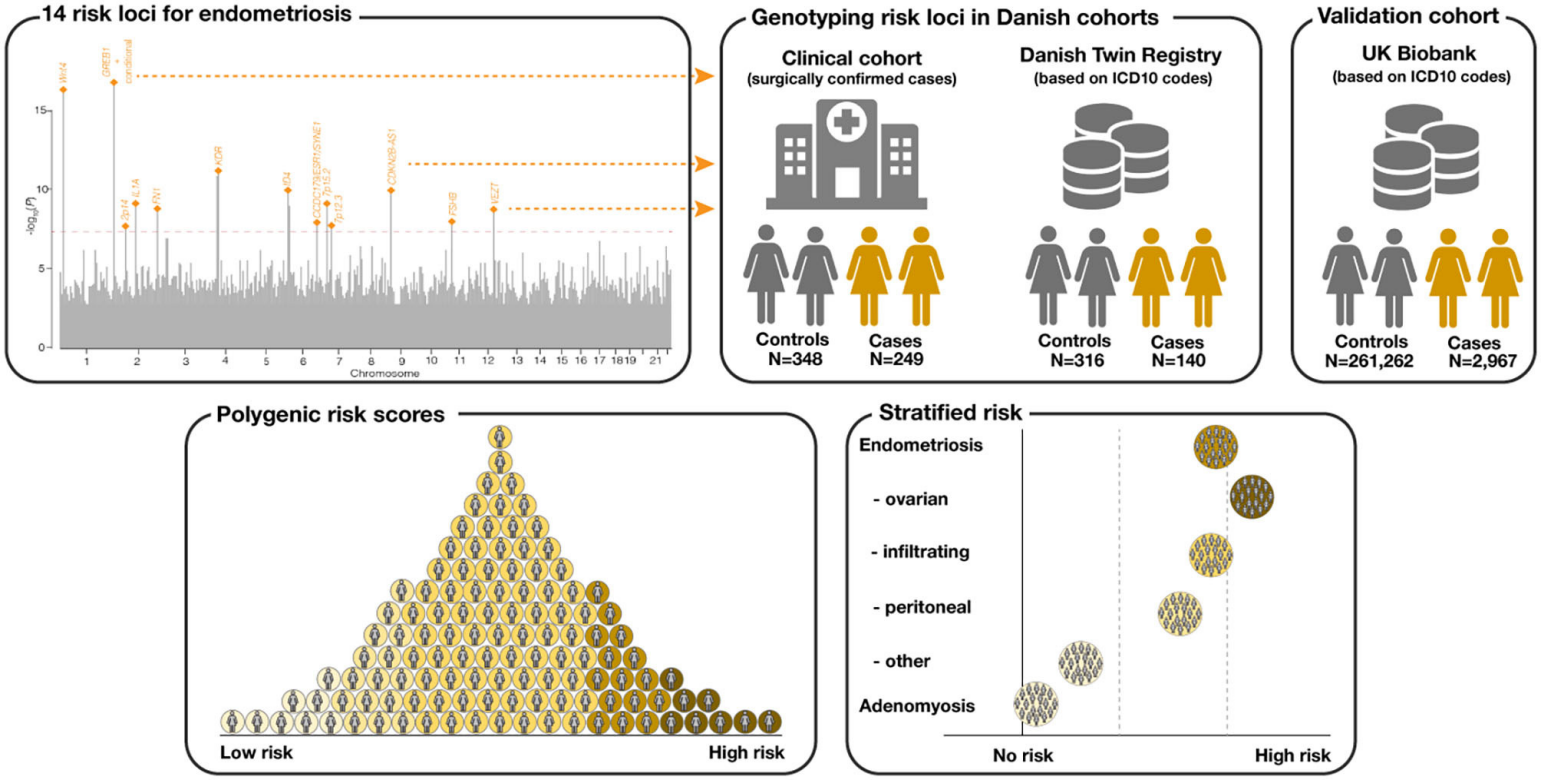
Conceptualization of the study. A total of 14 risk loci for endometriosis were identified from Sapkota et al. (9). Individuals from two Danish cohorts (a clinical cohort with surgically confirmed cases and a biobank cohort from the Danish Twin Registry (DTR) with cases and controls selected based on ICD-10 diagnosis codes) were genotyped for the 14 endometriosis risk variants. Unrelated, white British females from the UK Biobank served as an independent validation cohort. A 14 variant polygenic risk score (PRS) for endometriosis was computed for the Danish cohorts and the UK Biobank. Finally, the PRS was associated with different disease subtypes.

**Figure 2:**
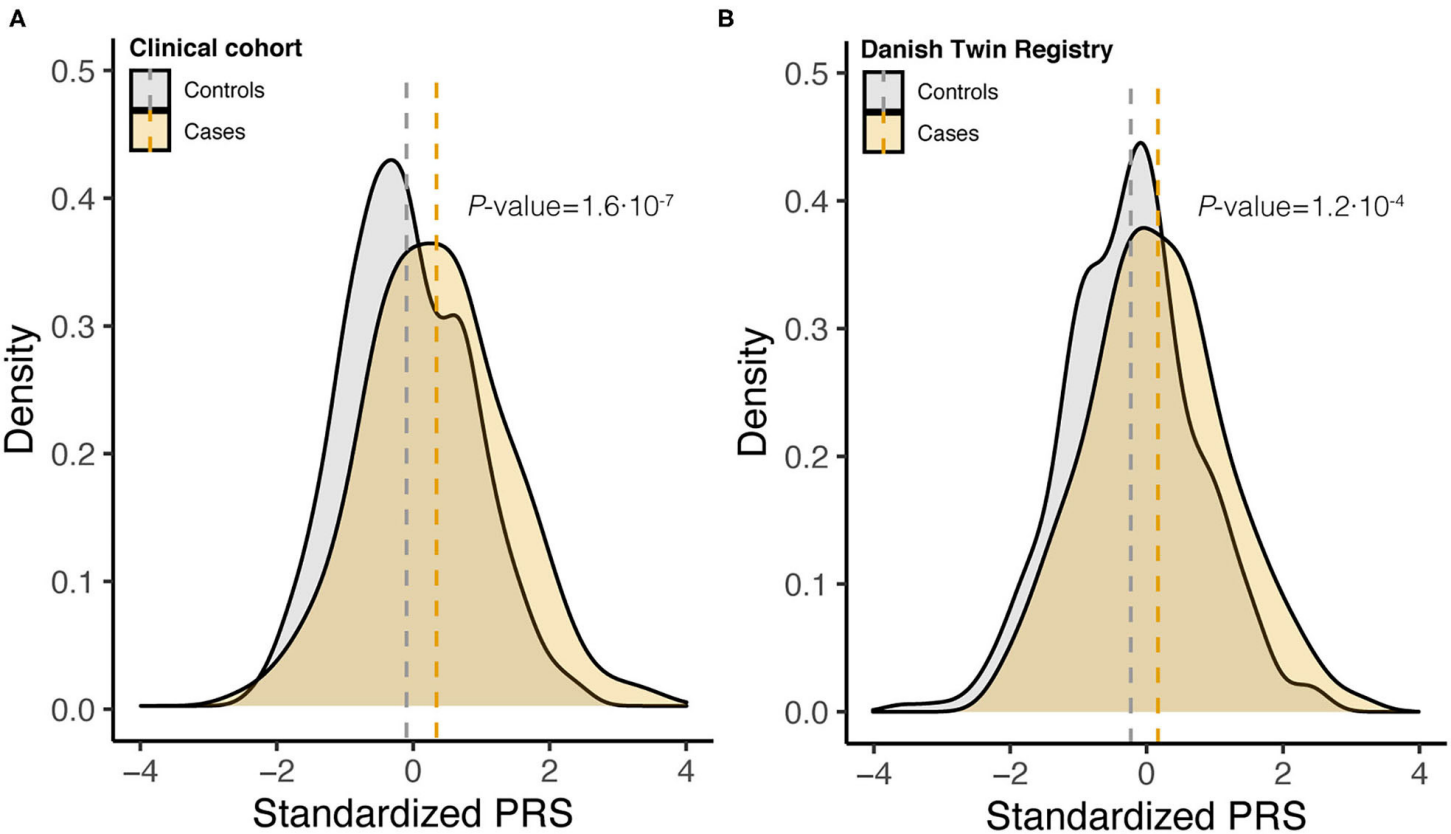
Density curves of standardized polygenic risk scores (PRS) stratified by case-control status for **(A)** clinical cohort (348 controls and 249 cases) and **(B)** DTR biobank cohort (316 controls and 140 cases). Vertical dashed lines indicate the within cohort sample mean, and *P*-values are from two-tailed Student's *t*-test.

To assess the joint effect of the 14 risk variants, we computed the PRS for endometriosis (Figure 1). For both Danish cohorts, the PRS distribution approximated a normal distribution, with the density plot for the cases shifting toward a higher PRS (Figure 2, Supplementary Table 3). The average standardized PRS was significantly higher in endometriosis cases compared with controls in both the clinical cohort (*P* = 1.6× 10^−7^) and within the DTR biobank cohort (*P* = 1.2·× 10^−4^; (Figure 2). The PRS significantly predicted endometriosis in surgically confirmed cases (OR) = 1.59, *P* = 2.57× 10^−7^, AUC = 0.68) and in cases ascertained from DTR biobank (OR = 1.50, *P* = 0.0001, AUC = 0.61). No significant difference was found in average PRS between cases within the two cohorts, indicating that biobank cases identified using ICD-10 codes and surgically confirmed cases have a comparable genetic burden (*P* = 0.15). In addition, no significant difference in average PRS between control samples in the two cohorts was found (*P* = 0.10). Based on this finding, we combined the clinical and DTR biobank cohorts (combined Danish cohort hereafter) in subsequent analyses.

**Figure 3:**
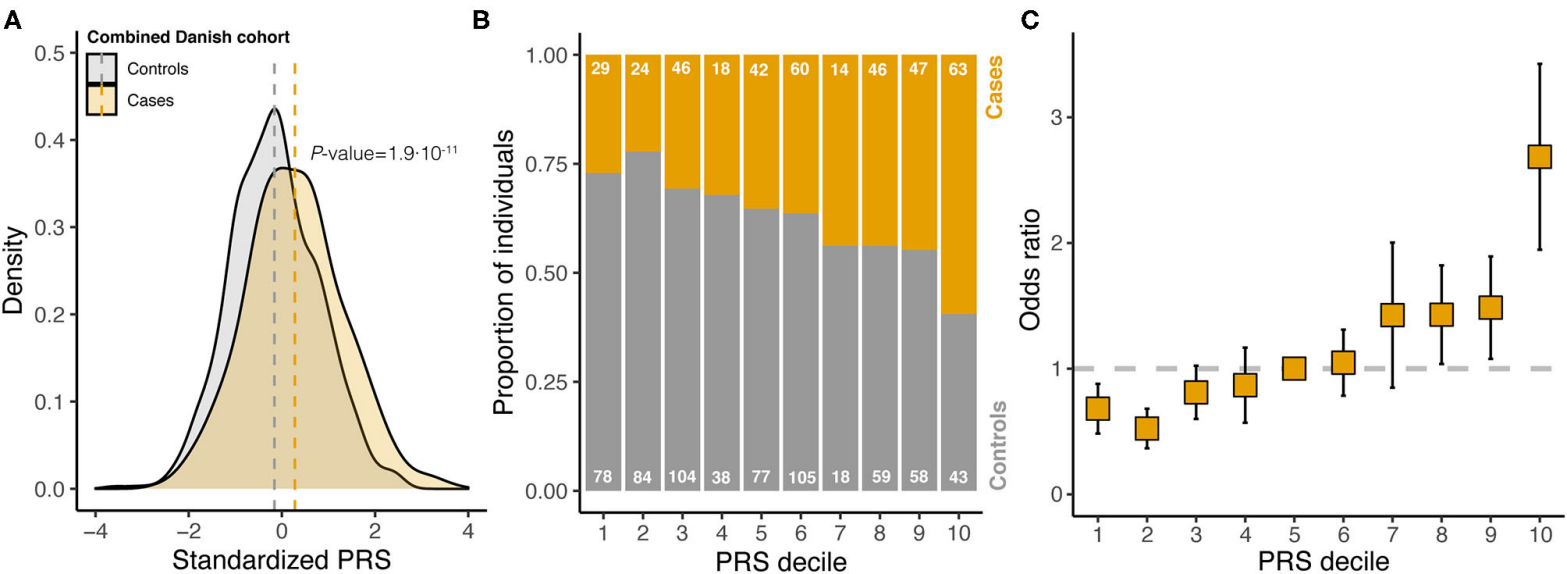
Analysis of standardized polygenic risk scores (PRS) in the combined Danish cohort. **(A)** Stratified by case-control status. Vertical dashed lines indicate the within cohort sample mean, and *P*-value is from a two-tailed Student's *t*-test. **(B)** PRS divided into deciles and the proportion of cases and controls are counted within each decile (numbers written on each bar). **(C)** For each decile, the odds ratio was estimated from logistic regression (error bars indicate standard error of the estimate; reference decile was set to decile 5). The dashed horizontal line indicates an odds ratio of 1, which is the reference level (decile 5).

In the combined Danish cohort, a significantly increased PRS was found for cases (mean 0.28, SD 1.04) compared with controls (mean −0.16, SD 0.94, *P* = 1.9·10^−11^) (Figure 3A). The discriminative ability of the 14-SNP PRS was AUC = 0.64 (Supplementary Table 4). Dividing the PRS into deciles, an increasing proportion of individuals with endometriosis was seen with increasing PRS (Figure 3B), with individuals in the top 10% decile having almost three times higher odds of disease compared with individuals with an average PRS (Figure 3C).

**Figure 4:**
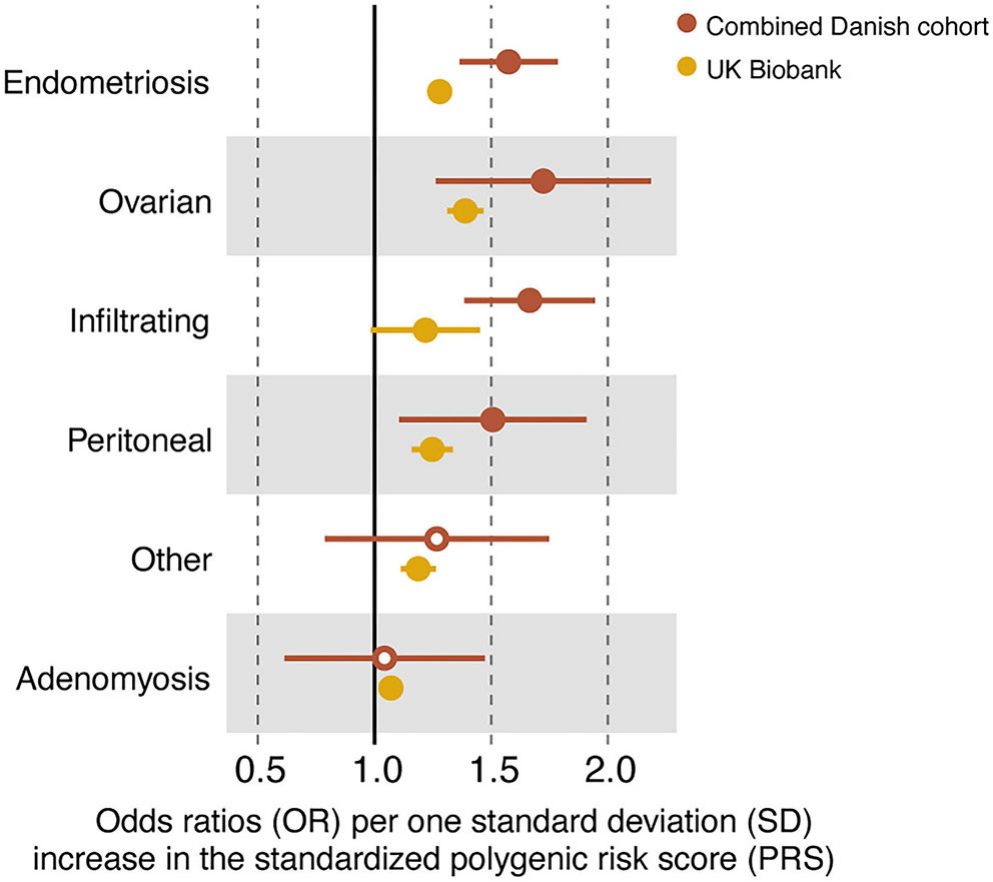
Forest plot of odds ratios (ORs) and their standard errors (error bars) computed for all endometriosis cases (N80.1–N80.9), the disease subtypes and adenomyosis within the combined Danish cohort and in the UK Biobank. Closed circles indicate a significant contribution of the standardized polygenic risk score (PRS) on case–control status (*P* < 0.05). See (Supplementary Table 4) for full statistical report.

To investigate how PRS possibly varied across endometriosis subtypes, the ICD-10 codes were collapsed into major subtypes: infiltrating, ovarian, peritoneal, and other (Table 1). Adenomyosis was included as a distinct entity. The prevalence of the different subtypes varied largely between the different cohorts (Table 1), e.g., with infiltrating lesions making up a large proportion of samples in the surgically confirmed group, but only a minor proportion in the other cohorts, reflecting that the surgically confirmed cases were collected at the specialized surgical unit in Denmark treating the most severe and complicated forms of endometriosis. A significant predictive ability for the PRS was found for all subtypes, except for “other” (Figure 4, Supplementary Table 4). The best AUC among the endometriosis subtypes was found for ovarian endometriosis (AUC = 0.64). These findings were replicated within the UK Biobank, again with best AUC for ovarian endometriosis (AUC = 0.60), demonstrating that ovarian endometriosis may have a higher genetic burden. Both in the combined Danish cohort and in the UK Biobank the 14-SNP PRS had no predictive power for adenomyosis (Figure 4, Supplementary Table 4).

## Discussion

Endometriosis has a strong genetic component (31–33), and genomic information could provide value if incorporated into a clinical decision support tool to identify women in need for laparoscopy. In this study, we constructed a PRS based on 14 established risk variants for endometriosis from the most recent published GWAS of endometriosis (9). We compared the PRS in two Danish case–control cohorts, representing two different and widely used designs, and found a comparable genetic burden between surgically confirmed cases and cases identified through health registries using ICD-10 codes. Since the mean PRS was not significantly different between cases in the two cohorts, our results suggest that ICD-10 codes are a reasonable tool to define cases for large GWAS studies or epidemiological studies of endometriosis.

Previously, it was shown that an increased genetic burden for endometriosis is associated with increasing severity (34, 35). As we did not have the severity score available to us, a formal replication of this finding was not possible. However, we believe our data are in good agreement with the previous finding as our surgically confirmed cases, ascertained at a surgical center specializing in severe and complicated endometriosis, had a slightly higher mean PRS than the remaining cases, although this was not statistically significant.

The finding of an AUC of around 0.64% for endometriosis is in line with other studies of PRS prediction in common complex diseases. Marigorta et al. (36) compared the predictive power of PRS for multiple common complex diseases and found that AUCs generally increase in the beginning when adding more SNPs to the PRS, but that the power of PRS seems to plateau around an AUC of ~60%. Due to the largely overlapping PRS distributions in cases and controls, and the relatively low discriminative accuracy of the PRS, it is not expected that the PRS alone will be useful for diagnostic or population screening purposes. It is, however, expected that genetics will become important for risk stratification in the future, when combined with information provided by other risk factors (37).

We observed in our study a lower effect size of the PRS in the UKB compared with the combined Danish cohort. A similar reduced effect size has been described for other variants in the UKB (38). This is likely because the UKB is enriched for healthy individuals, the so-called “healthy volunteer” selection bias (39), leading to an underestimation of penetrance (38). Despite the lower effect size all findings were replicated, demonstrating that a PRS has predictive abilities even in healthy populations.

Interestingly, we found that patients with adenomyosis had the same average PRS as the controls. This was observed in the combined Danish cohort and replicated in the UK Biobank. Endometriosis and adenomyosis represent different diseases but seem to share some pathophysiological pathways related to the junctional zone (40). Also, patients with severe endometriosis have a higher risk of adenomyosis (41). However, it is unknown if there are shared risk genes between the two conditions. Using the combined information from 14 risk SNPs for endometriosis, we found no discriminative ability for women with adenomyosis without a diagnosis of endometriosis, supporting the clinical observations that adenomyosis is different from endometriosis (26).

A limitation of this study is that the subjects were not genotyped using SNP array, and therefore we were not able to test if the predictive ability could be improved by including additional SNPs below the genome-wide significance threshold or correct for hidden population structure on a genome-wide scale. As ethnicity was not available to us in the two Danish cohorts, we cannot exclude that these cohorts had a minor proportion of non-European individuals. In previous studies, it has been shown that polygenic scores derived from a European GWAS had lower predictive power in other populations (42). If this is the case, a small proportion of non-European individuals would have resulted in lower discriminative ability in our study making the odds ratios a conservative estimate. As we replicated the results in the UKB using only white-British individuals, we find it unlikely that our results are driven by hidden population structures in the two Danish cohorts. Another limitation is the use of imputed SNPs in the UKB validation set, which introduces the potential for some incorrect genotype calling. Because all SNPs had high info score (above 0.9), we find it unlikely that this has affected the results significantly.

As sample sizes for GWAS grow, more and more genome-wide significant loci are being discovered for endometriosis, which could be incorporated into a genome-wide polygenic score. Also, more sophisticated methods are being developed (and already exist) for calculating polygenic risk scores that include millions of variants below the genome-wide significant level (43) and from correlated traits (44, 45). The scope of our work was to test a simple PRS to provide a conservative estimate on what benefits can be achieved by including genetic variant. Taken together our results suggest that genetic risk variants could be an important component of a composite risk stratification tool aimed at women with symptoms of endometriosis to reduce diagnostic delay.

In summary, we found that a polygenic score composed of as little as 14 SNPs is associated with endometriosis across all anatomical sites. Our study is a first important step to investigate the value of using common genetic variants to predict endometriosis.

## Data Availability Statement

The standardized PRS for the combined Danish cohort can be found within Supplementary Table S5. Researchers can gain access to the UKB data by applying to www.ukbiobank.ac.uk.

## Ethics Statement

The studies involving human participants were reviewed and approved by The Central Denmark Region Committees on Health Research Ethics and The Regional Committees on Health Research Ethics for Southern Denmark. The patients/participants provided their written informed consent to participate in this study.

## Author Contributions

MNye and LC planned the study. AF, RS, and LC was responsible for ascertainment of the patients and controls. RS, KK-M, and ST extracted DNA. KK-M and ST genotyped the samples and performed quality control. KK-M, ST, MNyg, CD, and PDR performed the statistical analysis. DR provided advise on the Danish health registries. MNye, MO, and LC supervised the study. All authors interpreted the results. MNye, KK, and PDR drafted the manuscript. All authors critically revised the manuscript, read, and approved the final manuscript.

## Funding

This study was supported by the Novo Nordisk Foundation by a grant to MNye (NNF21OC0071050) and funding for open access was obtained from support from Aarhus University.

## Conflict of Interest

The authors declare that the research was conducted in the absence of any commercial or financial relationships that could be construed as a potential conflict of interest.

## Publisher's Note

All claims expressed in this article are solely those of the authors and do not necessarily represent those of their affiliated organizations, or those of the publisher, the editors and the reviewers. Any product that may be evaluated in this article, or claim that may be made by its manufacturer, is not guaranteed or endorsed by the publisher.

## Supplementary Material

Supplementary Figure 1 Density curves of standardized polygenic risk scores (PRS) stratified by case-control status for UK Biobank (256,222 controls and 2,967 cases). Vertical dashed lines indicate the within cohort sample mean, and *P*-values are from two-tailed Student's *t*-test.

Supplementary Table 1 Single SNP association for the 14 SNPs with endometriosis (N80.1-80.9) in Danish clinical and DTR cohorts, within the UK Biobank, and from the Sapkota et al. (9) GWAS.

Supplementary Table 2 Primer sequences for the Fluidigm genotyping assay.

Supplementary Table 3 Mean and standard deviation (SD) of the standardized polygenic risk score (PRS) for each Danish cohort, the combined Danish cohort and within UK Biobank.

Supplementary Table 4 Discriminative power of the standardized polygenic risk score (PRS) within the Danish and UK Biobank for endometriosis (N80.1–N80.9), the disease subtypes, and adenomyosis (N80.0).

Supplementary Table 5 The standardized PRS for the combined Danish cohort.
