# Supplementary figures and images for: Polygenic Risk Score Prediction for Endometriosis
Source: Front Reprod Health. 2021 Dec 17;3:793226. doi: 10.3389/frph.2021.793226 (PMC9580817; doi:10.3389/frph.2021.793226)

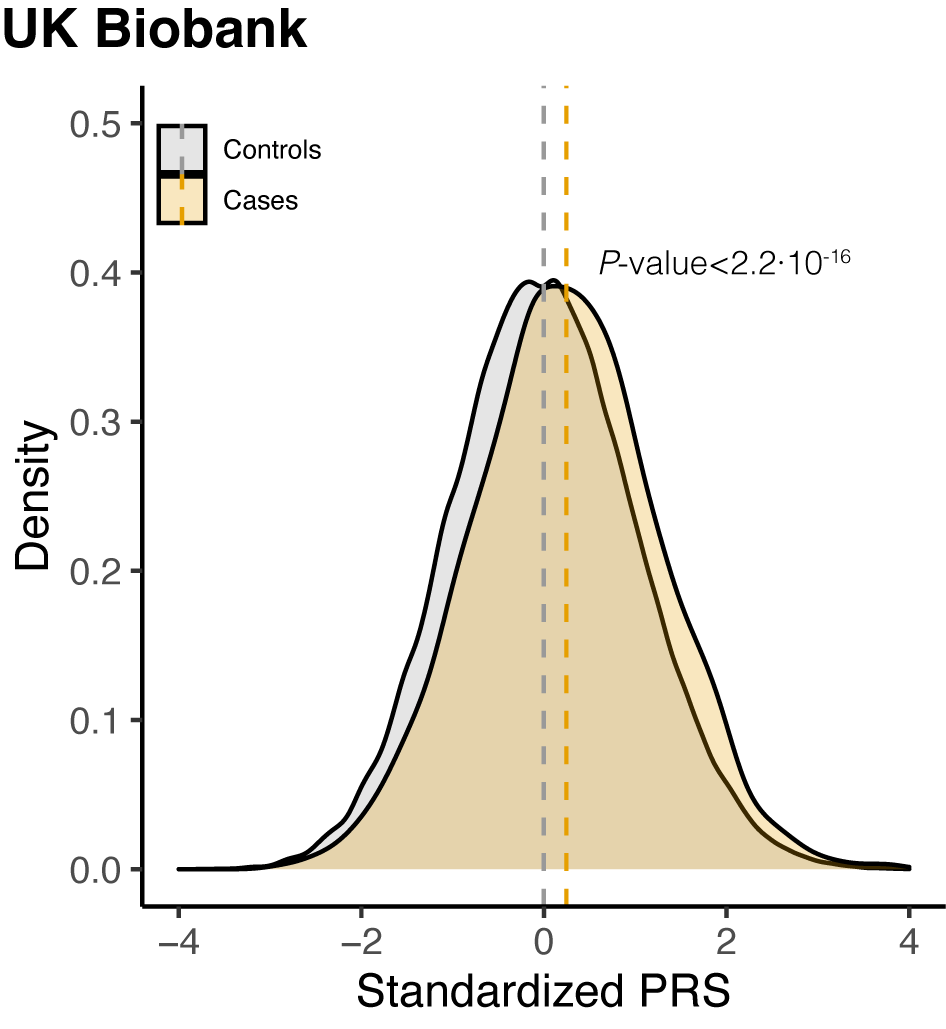

Supplement: Supplementary Figure 1 — Density curves of standardized polygenic risk scores (PRS) stratified by case-control status for UK Biobank (256,222 controls and 2,967 cases). Vertical dashed lines indicate the within cohort sample mean, and P-values are from two-tailed Student's t-test. [file Image_1.TIF]
