# Supplementary material for: Polygenic Risk Score Prediction for Endometriosis
Source: Front Reprod Health. 2021 Dec 17;3:793226. doi: 10.3389/frph.2021.793226 (PMC9580817; doi:10.3389/frph.2021.793226)
Supplement: Supplementary Table 2 — Primer sequences for the Fluidigm genotyping assay. [file Table_2.docx]

**Supplementary Table S2.** Primer sequences for the Fluidigm genotyping assay. All sequences are listed in 5’-3’ direction. ASP; allele-specific primer, LSP; locus-specific primer.

| **SNP** | **A1** | **A2** | **ASP1_SEQ (5’- 3’)** | **ASP2_SEQ (5’- 3’)** | **LSP_SEQ (5’- 3’)** |
| --- | --- | --- | --- | --- | --- |
| rs10167914 | A | G | CCTGTCAGACCACCTGACA | CCTGTCAGACCACCTGACG | GGCCTCGTGCCCATGCTA |
| rs11674184 | T | G | TCAGTTATCACCTCCTCCAGGAA | CAGTTATCACCTCCTCCAGGAC | TGCTGAGGCTCGTCTGCT |
| rs12037376 | A | G | GAGACCACAGGCTTCCATAT | GAGACCACAGGCTTCCATAC | GGTGGTTACGGGACTGCCT |
| rs1250241 | A | T | GCATGCTTTGGTAACTTCTGGAT | GCATGCTTTGGTAACTTCTGGAA | TGATATGTTTTTCTATTTATGTAGTCTTCTCTGAAGATGC |
| rs12700667 | A | G | AATATATTGAGAGTGAAAATGTGACAAAAGTGAA | TTGAGAGTGAAAATGTGACAAAAGTGAG | TGGTTGCATGTGTGGTCCGA |
| rs1537377 | C | T | CTCTTGGATTTGGGCAAGTGATAC | ACTCTTGGATTTGGGCAAGTGATAT | CAGATTTAAAAGCTGAGTCACAAAGAGTTTACTT |
| rs1903068 | A | G | AACACCAAGAGTGAACCCTCA | ACACCAAGAGTGAACCCTCG | CCTACACTGACACATCATTATTATCCAAATTCCA |
| rs1971256 | T | C | GAAGATCTGGCTGGCTCAGT | AGATCTGGCTGGCTCAGC | TGGGCCCCGATGCCAA |
| rs4762326 | C | T | AGTGAATTTACTTTAATTGGAGGCCAAC | GATAAGTGAATTTACTTTAATTGGAGGCCAAT | TGTTGAAACAAATGGCTAGTTATAAAATCACTCAC |
| rs6546324 | A | C | CCATTTACCAAATTGAAAATCTGAGCCTT | CATTTACCAAATTGAAAATCTGAGCCTG | CCCCTTACTAGCTATGGAGCTGT |
| rs71575922 | C | G | AAAAGCAATCATTTAAAATCTTTGTATATCAACTTC | AAAAGCAATCATTTAAAATCTTTGTATATCAACTTG | GCAGGAACCAACCCACGTT |
| rs74485684 | C | T | GTTCTCAAGCAATCTTCAGGTAATGC | TGTTCTCAAGCAATCTTCAGGTAATGT | CCTCAGAGCAATCTTTTAAGGTGGGA |
| rs74491657 | A | G | CCTCCGACCTGCCCT | CCTCCGACCTGCCCC | AGTGTGTGCCTGTGTGCAGA |
| rs77294520 | C | G | AGGTGCCGGGCTTTAGC | AGGTGCCGGGCTTTAGG | GGAAAGCAGGATAATGGCTCCC |
