# Supplementary material for: Polygenic Risk Score Prediction for Endometriosis
Source: Front Reprod Health. 2021 Dec 17;3:793226. doi: 10.3389/frph.2021.793226 (PMC9580817; doi:10.3389/frph.2021.793226)
Supplement: Supplementary Table 3 — Mean and standard deviation (SD) of the standardized polygenic risk score (PRS) for each Danish cohort, the combined Danish cohort and within UK Biobank. [file Table_3.docx]

**Supplementary Table S3.** Mean and standard deviation (SD) of the standardized polygenic risk score (PRS) for each Danish cohort, the combined Danish cohort and within UK Biobank. P-values from Student’s t-test comparing mean PRS for Danish endometriosis cases and controls, respectively, are shown (comparisons are highlighted in grey).

| **Cohort** | **Standardized PRS** | | ***P*-value** |
| --- | --- | --- | --- |
|  | **Mean** | **SD** |  |
| ***Combined Danish cohort*** |  |  |  |
| - Cases, clinical | 0.34 | 1.05 | 0.15 |
| - Cases, DTR | 0.18 | 1.02 |  |
| - Cases, combined | 0.28 | 1.04 |  |
| - Controls, clinical | -0.10 | 0.91 | 0.10 |
| - Controls, DTR | -0.22 | 0.97 |  |
| - Controls, combined | -0.16 | 0.94 |  |
| ***UK Biobank*** |  |  |  |
| - Cases | 0.25 | 1.01 |  |
| - Controls | -0.006 | 1.00 |  |
