# Supplementary material for: Polygenic Risk Score Prediction for Endometriosis
Source: Front Reprod Health. 2021 Dec 17;3:793226. doi: 10.3389/frph.2021.793226 (PMC9580817; doi:10.3389/frph.2021.793226)
Supplement: Supplementary Table 4 — Discriminative power of the standardized polygenic risk score (PRS) within the Danish and UK Biobank for endometriosis (N80.1–N80.9), the disease subtypes, and adenomyosis (N80.0). [file Table_4.docx]

**Supplementary Table S4.** Discriminative power of the standardized polygenic risk score (PRS) within the Danish and UK Biobank cohorts for endometriosis (N80.1-N80.9), the disease subtypes, and adenomyosis (N80.0). Table contains number of cases within subgroup (*n*), estimated odds ratio (OR), the associated standard error of the OR estimate (SE), *P*-value from logistic regression of whether PRS significantly contribute to the discrimination of cases and control (*P*), Nagelkerke’s $R_{Nag}^{2}$￼), and area under the receiver operating characteristic curve (AUC).

|  | **Combined Danish cohort** | | | | | |  | **UK Biobank** | | | | | |
| --- | --- | --- | --- | --- | --- | --- | --- | --- | --- | --- | --- | --- | --- |
| **Case category** | ***n*** | **OR** | **SE** | ***P*** | $\boldsymbol{R}_{\boldsymbol{Nag}}^{\boldsymbol{2}}$ | **AUC** |  | ***n*** | **OR** | **SE** | ***P*** | $\boldsymbol{R}_{\boldsymbol{Nag}}^{\boldsymbol{2}}$ | **AUC** |
| Endometriosis | 389 | 1.57 | 0.11 | 2.5·10^-11^ | 0.061 | 0.64 |  | 2,967 | 1.28 | 0.02 | <2.2·10^-16^ | 0.006 | 0.57 |
| - infiltrating^*^ | 210 | 1.66 | 0.14 | 2.7·10^-9^ | 0.065 | 0.60 |  | 105 | 1.22 | 0.12 | 0.04 | 0.002 | 0.55 |
| - ovarian^†^ | 75 | 1.72 | 0.24 | 6.7·10^-5^ | 0.051 | 0.64 |  | 1,158 | 1.39 | 0.04 | <2.2·10^-16^ | 0.009 | 0.60 |
| - peritoneal^‡^ | 60 | 1.51 | 0.21 | 2.6·10^-3^ | 0.026 | 0.61 |  | 736 | 1.25 | 0.04 | 5.7·10^-10^ | 0.004 | 0.56 |
| - other^**^ | 44 | 1.27 | 0.25 | 0.22 | 0.008 | 0.53 |  | 968 | 1.19 | 0.04 | 7.5·10^-8^ | 0.002 | 0.55 |
| Adenomyosis | 25 | 1.04 | 0.22 | 0.85 | 0.000 | 0.50 |  | 1,883 | 1.07 | 0.02 | 0.003 | 0.000 | 0.52 |
| ^*^ N80.4, N80.5  ^†^ N80.1  ^‡^ N80.2, N80.3  ^**^ N80.6, N80.8, N80.9 | | | | | | | | | | | | | |
